# 

**Published:** 1892-01

**Authors:** 


					﻿ESTABLISHED 1066.	SUBSCRIPTION, $2.00.
/ArnrC^^^tfrAi
EDITED BY
H. V. M. MILLER, M. D., LL. D. L. P. KENNEDY, A. M., M. D.
VIRGIL O. HARDON, M. D.	M. B. HUTCHINS, M. D.
F. W. McRAE. M. D.	L. B. GRANDY, M. D.
Published by JAS. P. HARRISON & CO., Atlanta, Ga.	I
Entered at the Post-Office at Atlanta, Ga., as second-class matter.
vol. vul ! Atlanta, Ga., January, 1892. No. 11.
				

